# Supplementary material for: Splice-Junction-Based Mapping of Alternative Isoforms in the Human Proteome
Source: Cell Rep. Author manuscript; Available in PMC 2020 Jan 15. (PMC6961840; doi:10.1016/j.celrep.2019.11.026)
Supplement: 3 [file NIHMS1546469-supplement-3.zip › DF2/PXD009737/TestisGluC-91-Q86YF9-QPAAPAVPMNAPALHTLETKSSLPMVHGRE.pdf]

A

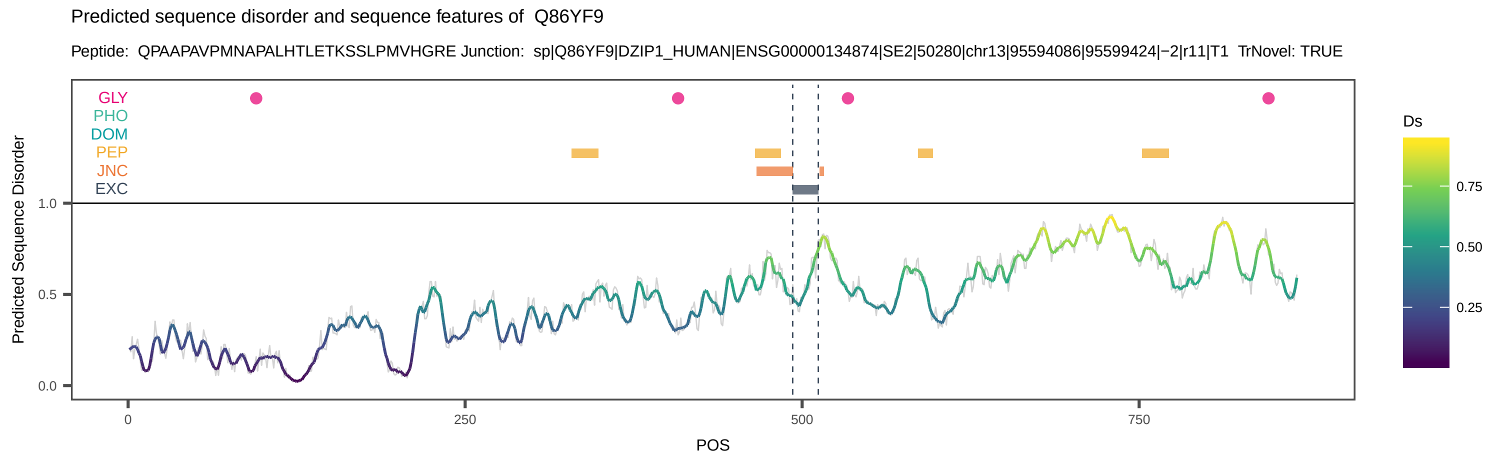

Predicted sequence disorder and sequence features of Q86YF9
Peptide: QPAAPAVPMNAPALHTLETKSSLPMVHGRE Junction: sp|Q86YF9|DZIP1_HUMAN|ENSG00000134874|SE2|50280|chr13|95594086|95599424|−2|r11|T1 TrNovel: TRUE
GLY
PHO
DOM
PEP
JNC
EXC
Predicted Sequence Disorder
1.0
0.5
0.0
0
250
500
750
POS
Ds
0.75
0.50
0.25

B

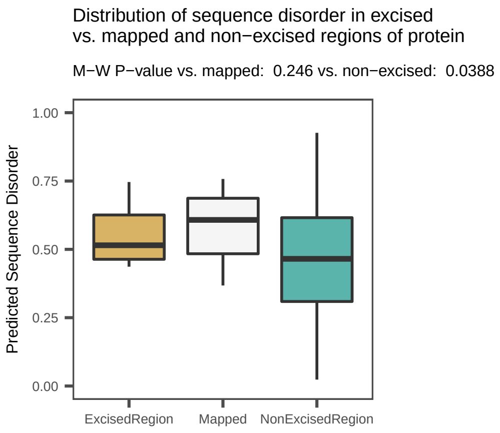

Distribution of sequence disorder in excised
vs. mapped and non−excised regions of protein
M−W P−value vs. mapped: 0.246 vs. non−excised: 0.0388
Predicted Sequence Disorder
1.00
0.75
0.50
0.25
0.00
ExcisedRegion
Mapped
NonExcisedRegion

C
